# Surface evolution of manganese chloride aqueous droplets resulting in self-suppressed evaporation

**DOI:** 10.1038/srep13322

**Published:** 2015-08-21

**Authors:** Xiping Zeng, Yifan Zhang, Zengzilu Xia, Li Wang, Cong Wang, Yingzhou Huang, Rong Shen, Weijia Wen

**Affiliations:** 1 Nano Science and Technology Program / Department of Physics, Hong Kong University of Science and Technology, Clear Water Bay, Kowloon, Hong Kong; 2 Institute of Chemistry, the Chinese Academy of Sciences, Zhongguancun North 1st St, Haidian, Beijing, China; 3 Chongqing University, No. 174 Shazhengjie, Shapingba, Chongqing, 400044, China; 4 Institute of Physics, Chinese Academy of Sciences, Haidian, Beijing, China

## Abstract

The exchange kinetics of liquid water, which are of fundamental interest and have potential applications, remain unclear. A fantastic and extraordinary phenomenon was observed during the evaporation of a water droplet doped with manganese chloride. As observed from the evolution of this type of droplet, a thin film was formed on the surface with an exothermic phase transition, resulting in self-suppressed evaporation. The MnCl_2_-doped water droplets were maintained in a relative humidity (RH) of 50% at 40 °C for more than a week and for longer than two months at a temperature of 25 °C. In contrast, a pure water droplet can only be sustained for a few minutes. The self-suppressed evaporation of doped water may be due to the special hydration of the accumulated manganese and chloride ions at the surface, decreasing the surface tension.

The evaporation of sessile droplets is important in a large number of areas including heat-transfer applications, printing, washing, coating, and foliar fertilizer delivery. The transport properties of a liquid, especially the evaporation rate, are of great interest in the assessment of hazards due to volatile chemicals and the release of volatile active species, such as perfumes and flavours, in water and soil conservation. However, very few studies have been reported on the evaporation of water. The evaporation rate of a water droplet depends on various factors including the temperature, relative humidity, substrates and additives, such as surfactants and electrolytes^1^,^2^.

During the past few decades, the study of the structures and behaviours of ions at water surfaces has received considerable interest^3^,^4^,^5^,^6^. In addition to the previously mentioned practical reasons, the complexity of the structures of water and the many ion hydration types provides its own motivation for researchers. The distribution of soluble ions at the air/water interface is important for understanding the kinetics and dynamics of the evaporation process and interfacial reactions. The traditional thermodynamic model suggests that the ions in water should be repelled from the air/water interface. In contrast, recent molecular dynamics simulations predicted that some simple ions (e.g., I^−^ and Br^−^) might accumulate at the water surface^7^,^8^. Recently, due to the development of phase-sensitive nonlinear optical techniques, such as second harmonic generation^9^,^10^,^11^, vibrational sum-frequency generation^12^,^13^,^14^ and photoelectron spectroscopy in liquid jets^15^,^16^,^17^, researchers have discovered that aqueous surfaces are not as passive as classical theory predicted, which suggested that aqueous surfaces would be essentially ion free and similar to the surface of neat water. Small hard ions, such as alkali metal cations or fluorine ions, are repelled from the surface. However, other inorganic ions, such as soft anions (heavier halides), hydronium, ammonium and nitrate, exhibit a relative propensity for the surface. The anionic size, solvent polarizability, and hydrogen bonding pattern may be possible driving forces^13^,^18^,^19^,^20^,^21^. However, the driving forces are a controversial issue, and some experimental results have contradicted the predictions from MD simulations^22^.

Surprisingly, two different types of evaporation were observed for aqueous electrolyte droplets, which have not been previously reported. In room temperature with 50 ± 5% RH, a 20-μl pure water sessile droplet could only exist for approximately 30 minutes. However, a droplet of the same size of 0.5 M sodium chloride was maintained for a slightly longer period of time, and the sodium chloride precipitated during the evaporation process. However, a droplet of the same size of 0.5 M manganese chloride was stable for more than two months, and a thin film was formed on the surface of the droplet, which suppressed the evaporation of the droplet. These phenomena not only provide remarkable insight into moisturizing and saving water but also have significant potential in studies of the hydration and distribution of ions in water.

## Results and Discussion

The evaporation of a droplet is a very complicated process that is affected by various factors, such as the ambient temperature, RH, vapour diffusion, and the contact angle of the sessile droplet. The additive should be the most important factor. To avoid interferences from other factors, the conditions in the parallel experiments were the same except this mentioned one.

**Figure 1:**
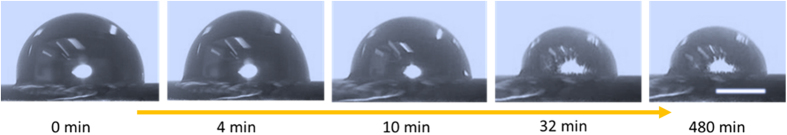
Evaporation process of a water droplet with 0.5 M manganese chloride. The scale bar is 2 mm.

The cleaned glass slides modified with n-decyltrimethoxysilane via chemical vapour deposition were used as the substrates, and the contact angle of pure water on this substrate was 90 ± 3°. The RH was maintained at 50 ± 5% in all of the experiments. The entire evaporation process was recorded using a homemade horizontal optical system and a commercial CCD. When the substrate temperature was maintained at 40 °C, a 20-μl droplet dries up in approximately 30 minutes, which is a common phenomenon. A water droplet with the same volume containing 0.5 M sodium chloride was sustained slightly longer for approximately 40 minutes, which was predicted based on classical Raoult’s law reported in 1882. Surprisingly, the droplet existed for more than one week when it contained 0.5 M manganese chloride. In addition, the droplet could be maintained for more than two months when stored at room temperature (25 °C). However, a pure water droplet and a sodium chloride droplet dry up in two hours under the same conditions. As shown in Fig. 1, the manganese chloride droplet (20 μl) shrank on the substrate in the initial stage, and the shape of the droplet did not change after approximately 30 minutes.

**Figure 2:**
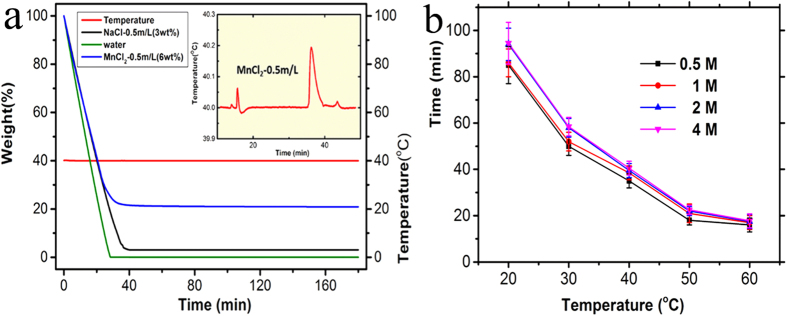
TGA test. (**a**) The comparison of the three types of water droplets, 0.5 M sodium chloride aqueous solution and 0.5 M manganese chloride aqueous solution, when temperature was maintained at 40 °C. The inset shows the relationship between the temperature and time for the evaporation of the manganese chloride droplet. (**b**) the relationship between phase transition time and the concentration of the manganese chloride under different temperature.

To validate the observed phenomena, thermogravimetric analysis (TGA) was used to determine the thermal stability and evaporation process of the three types of droplets. All of the measurements were performed in air to simulate the situations that occur in the natural environment. With the temperature maintained at 40 °C, the proportion of retained weight was recorded as a function of increasing time. As shown in Fig. 2a, the droplet of pure water volatilized in approximately 30 minutes, and for the volatilization of the droplet with 0.5 M sodium chloride, only sodium chloride remained after approximately 40 minutes. However, the droplet containing 0.5 M manganese chloride continuously shrank in the beginning but did not exhibit obvious changes after approximately 38 minutes, and approximately 22% of its weight remained, which is much greater than the weight of the manganese chloride contained in the droplets. These results indicated that a significant volume of water was still present.

Scanning differential calorimetry was also performed to study the evaporation process. The inset in Fig. 2a shows the change in the temperature as the time increased for the evaporation process of the manganese chloride droplet. Two exothermic peaks were observed at 18 minutes and 38 minutes, which are most likely due to exothermic phase transitions. This phenomenon was not observed during the evaporation process of the other two types of droplets. There are an obvious turning points on the TGA curve of the manganese chloride droplets at 38 minutes when the phase transition finished. We found that the phase transition time decreases as the temperature increases (Fig. 2b). Although the droplet are finally dried up, there are still an obvious turning points on the TGA curves when temperature was maintain at 50 °C and 60 °C. While the phase transition time is a little longer when the concentration of the manganese chloride raised, which could be due to the hydration of ions and their redistribution at the surface.

**Figure 3:**
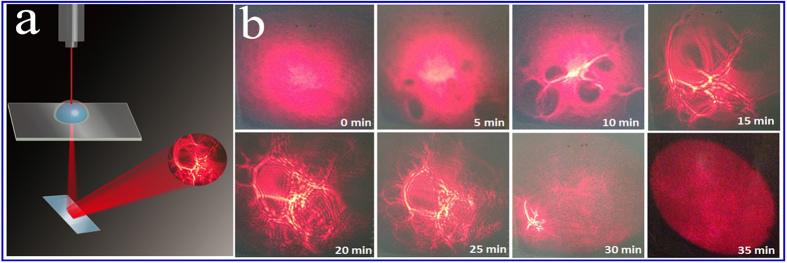
(**a**) Schematic representation of the homemade apparatus for the observation of the surface evolution in the manganese chloride droplet evaporation process. (**b**) Evolution of the diffraction patterns.

**Figure 4:**
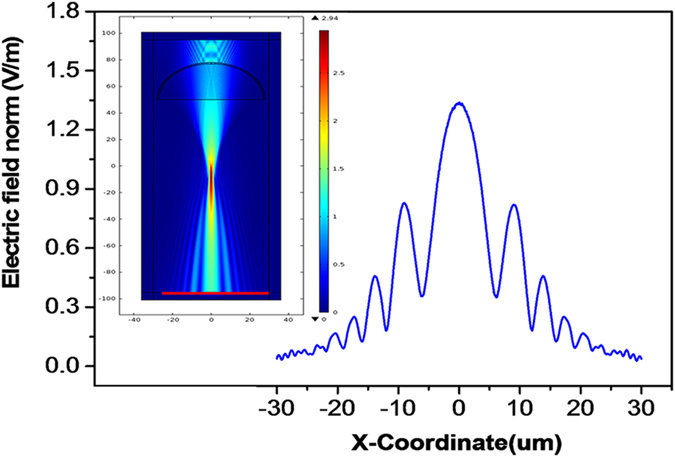
COMSOL multiphysics simulation of the diffraction pattern of the laser due to the thin film on the droplet. The inset shows the 2D distribution of the electric field. The line graph is the distribution of the electric field on the line, which is indicated by the red line in the inset.

Inspired by the study of Newton’s ring and interference fringes formed with curved thin sheets^23^,^24^, a creative homemade light apparatus was designed to monitor the surface evolution of the droplet with manganese chloride. As shown in Fig. 3a, a 20-μl droplet was dropped onto a modified glass slide mounted on a heating system with the temperature was maintained at 40 °C. A 633 nm laser beam with a diameter of approximately 1 mm vertically penetrated through the centre of the droplet and then was reflected on a vertical screen with a reflector. The patterns of the laser on the screen were recorded by a commercial CCD (Fig. 3a). After several minutes of evaporation, some interesting patterns began to appear, as shown in Fig. 3b. The interference patterns were easily identified at approximately 15 minutes. The irregular patterns evolved as the evaporation progressed, which may be due to some small thin film fragments being formed and fluctuating on the surface of the droplet. After approximately 20 minutes, the film fragments joined together, and the distance of the interference fringes began to decrease. Therefore, the thickness of the film continued to increase. The interference fringes became obscure and finally disappeared after approximately 35 minutes, which indicated that the thickness was too large to produce interference or there was no clean-cut difference between the film and the bulk of the droplet. A simple simulation was performed with COMSOL Multiphysics to confirm the diffraction phenomena due to the curved thin film. The model, which was based on the Wave Optics module, was scaled down by 50 times compared to the actual situation due to computational complexity. The film thickness was 1 μm, and its index of refraction was 1.4. In addition, the diffraction patterns are shown in Fig. 4.

**Figure 5:**
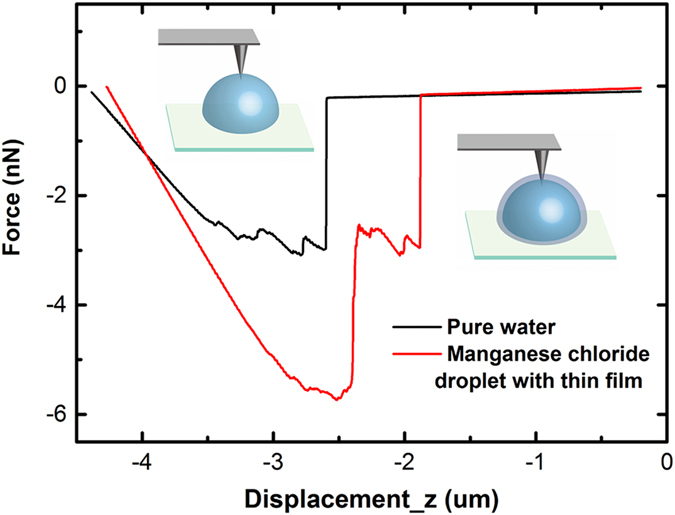
Investigation of the surface of the droplets using AFM. An AFM tip was inserted approximately 2 μm into the surface of the droplets and then drawn out. The relationship between the force and the vertical displacement was recorded.

AFM was also used to confirm the formation of the thin film on the droplet, as shown in Fig. 5. The droplet was pre-heated at 40 °C for approximately 35 minutes. Then, the AFM probe was pierced 2 μm into the droplet from the top and then slowly drawn out. The relationship between the drawing force and the vertical displacement was recorded. Based on the results for the pure water droplet, the force became more negative in the initial stage due to the adhesion force between the water and the tip, and then, the force suddenly returned to zero, which indicates that the water was detached from the tip surface. In contrast, there were two sudden changes observed in the plot for the manganese chloride droplet. The first change corresponded to the liquid beneath the film detaching from the tip, and the second one corresponded to the film detaching from the tips. Therefore, based on these results, the film thickness is approximately 1 μm, which is approximately equal to the width between the two steps.

The electrolyte typically precipitates at the bottom of the solution during evaporation, which is the same as that observed for the aqueous solution of sodium chloride. Therefore, the film that formed on the surface of the droplet in the evaporation process of the manganese chloride droplet and the self-suppressed evaporation are interesting phenomena.

**Figure 6:**
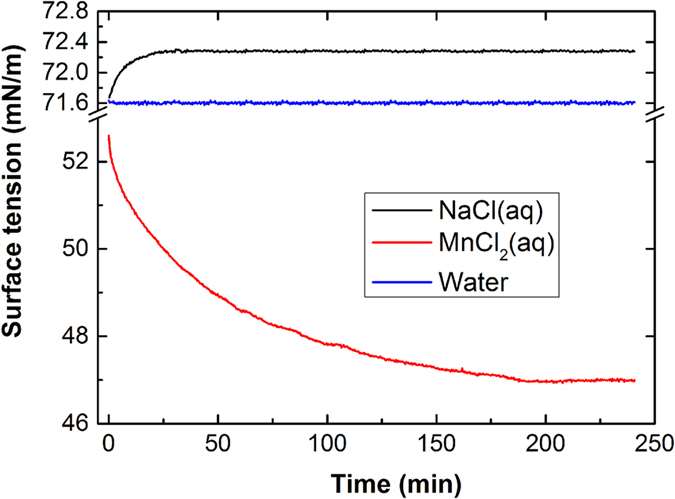
Evolution of the surface tension of the three types of liquids as a function of time using a 1 M sodium chloride aqueous solution, a 1 M manganese chloride aqueous solution, and pure water.

A modified setup was developed to monitor the variation in the surface tension as the time increased. A standard platinum plate was mounted on a highly sensitive balance system and immersed in a beaker with approximately 20 ml of a solution. The force was recorded and converted to surface tension with software by considering the geometrical dimensions of the platinum plate. The details of the surface tension are shown in Fig. 6 The surface tension of water was 71.6 mN/m and exhibited no obvious change with time. The surface tension of the sodium chloride aqueous solution increased during the initial 20 minutes and then remained stable. The increase in the surface tension of sodium chloride aqueous solution compared to that of pure water is consistent with previous studies^25^. Surprisingly, the surface tension of the manganese chloride solution is much smaller than the surface tension of water and exhibited a continuous decrease for approximately three hours prior to becoming stable. These phenomena may be due to the formation of a thin film on the surface of the manganese chloride solution. The manganese chloride tended to accumulate on the surface due to its ability to decrease the surface tension. The sodium chloride increased the surface tension, which resulted in its depletion from the surface and precipitation in the bottom of the droplet. The manganese chloride exhibits a stronger interaction with water molecules. Therefore, the aggregated manganese chloride formed a film with water to self-suppress the evaporation of the droplet. The film was metastable and exhibited no crystal structure because no X-ray diffraction (XRD) peaks were observed in the XRD experiment (PANalytical X’pert Pro). There was a large variation in the time required for the stabilization of the surface tension. For example, the surface tension of the sodium chloride aqueous solution stabilized in approximately 20 minutes, and the manganese chloride aqueous solution required approximately three hours. This result inspired a direct approach for determining the reorientation time of the water molecules, which typically requires complicated technology^26^,^27^,^28^.

**Figure 7:**
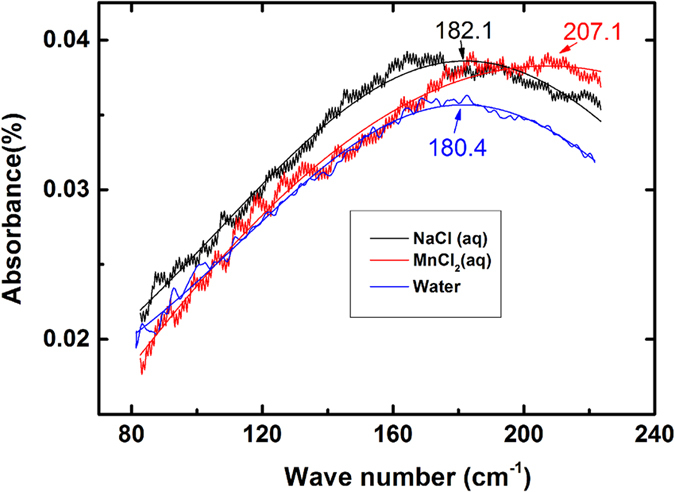
Far ATR-IR spectra with Gaussian fitting (the smooth line) for water as well as the sodium chloride aqueous solution and manganese chloride aqueous solution.

To determine the influence of ions on the hydration water, especially the hydrogen bond, far infrared spectra were adapted to investigate the binding. Because the molar absorptivity of water is too high to examine the solutions by common transmission spectroscopy, the application of an attenuated total reflection (ATR) unit was employed for the experiments. ATR affects the peak of the absorption spectra due to anomalous dispersion, resulting in decreased wavenumbers in the ATR spectrum compared to the absorption spectrum^29^,^30^. As shown in Fig. 7, the hydrogen bond stretching (181 cm^−1^) shifted by approximately 2 cm^−1^ and 27 cm^−1^ when sodium chloride and manganese chloride were added, respectively. The hydrogen bonds in the aqueous sodium chloride droplets are slightly stronger than those in pure water. Due to the more robust interaction between the water molecules in the sodium chloride droplets, the sodium chloride droplets were maintained for a slightly longer period of time than the pure water droplets. Manganese chloride caused a larger increase in the hydrogen bonds than sodium chloride, which indicates that the water molecules are held more strongly in the solution. In the evaporation process of the droplets with manganese chloride, the accumulating ions at the surface coordinate with water molecules, which interrupts the hydrogen bonds between the non-coordinated water^31^. Thus, the energy releases as shown in the inset of Fig. 2a, resulting in a decrease in the surface tension. Manganese bromide exhibited phenomena similar to those of manganese chloride due to the presence of the same cations, and the Br^−^ anions were even more prone to accumulating at the surface of the water than the Cl^−^ anions^25^,^32^.

## Methods

The RH was controlled by the central air conditioning with an ultrasonic humidifier (Air-O-Swiss AOS 7135) and recorded by a hygrometer (Oregon Scientific, THG 312) in all of the experiments. The TGA was performed using a TGA Q5000 with an airflow rate of 25.0 ml/min. The evolutions of the surface tensions of the three types of liquids were recorded by a highly sensitive microelectromechanical balance system (Kuess K100SF). The relationship between the force and the vertical displacement that is shown in Fig. 5 was acquired using AFM. An AFM (MFP-3D) with probes (HQ:CSC38/AL BS) with a spring constant (k) of 0.3 Nm^−1^ and a tip radius of 8 nm was used under ambient conditions. The XRD patterns were recorded on a PW1830 (Philips, Cu_kα_ radiation *λ* = 1.541874 Å). The Far FT-IR spectra were recorded on a Bruker Vertex 70 spectrometer with a Pike Gladi ATR unit equipped with a diamond at 45° as the internal reflecting element. The entire system was sealed and purged with nitrogen for 12 hours to prevent interference from the water molecules in the air.

## Additional Information

**How to cite this article**: Zeng, X. *et al*. Surface evolution of manganese chloride aqueous droplets resulting in self-suppressed evaporation. *Sci. Rep*. **5**, 13322; doi: 10.1038/srep13322 (2015).
